# Development of a multidecadal land reanalysis over High Mountain Asia

**DOI:** 10.1038/s41597-024-03643-z

**Published:** 2024-07-27

**Authors:** Fadji Z. Maina, Yuan Xue, Sujay V. Kumar, Augusto Getirana, Sasha McLarty, Ravi Appana, Bart Forman, Ben Zaitchik, Bryant Loomis, Viviana Maggioni, Yifan Zhou

**Affiliations:** 1 NASA Goddard Space Flight Center, Hydrological Sciences Laboratory, Greenbelt, Maryland USA; 2 University of Maryland, Baltimore County, Goddard Earth Sciences Technology and Research Studies and Investigations, Baltimore, Maryland USA; 3 Department of Geography and Geoinformation Science, George Mason University, Fairfax, VA USA; 4 Science Applications International Corporation, McLean, VA USA; 5 Washington State University, Pullman, Washington USA; 6 Department of Civil & Environmental Engineering, University of Maryland, College Park, MD USA; 7 Department of Earth and Planetary Sciences, Johns Hopkins University, Baltimore, MD USA; 8 Geodesy and Geophysics Laboratory, NASA Goddard Space Flight Center (GSFC), Greenbelt, MD USA; 9 Department of Civil, Environmental & Infrastructure Engineering, George Mason University, Fairfax, VA USA; 10 Present Address: Lynker at NOAA/NWS/NCEP/EMC, College Park, Maryland USA

**Keywords:** Hydrology, Hydrology

## Abstract

Anthropogenic and climatic changes affect the water and energy cycles in High Mountain Asia (HMA), home to over two billion people and the largest reservoirs of freshwater outside the polar zone. Despite their significant importance for water management, consistent and reliable estimates of water storage and fluxes over the region are lacking because of the high uncertainties associated with the estimates of atmospheric conditions and human management. Here, we relied on multivariate data assimilation (MVDA) to provide estimates of energy and water storage and fluxes that reflect the processes occurring in the region such as greening and irrigation-driven groundwater depletion. We developed and employed an ensemble precipitation estimate by blending different precipitation products thereby reducing the uncertainties and inconsistencies associated with precipitation in HMA. Then, we assimilated five variables that capture the changes in hydrology in response to climate change and anthropogenic activities. Overall, our results have shown that MVDA has allowed a better representation of the land surface processes including greening and irrigation-driven groundwater depletion in HMA.

## Background & Summary

High Mountain Asia (HMA) hosts the largest reservoirs of the freshwater outside the polar regions and stretches around 3900 km eastward and 2900 km northward. The region covers in various proportions 9 countries (China, Myanmar, Bhutan, Nepal, Bangladesh, India, Pakistan, Afghanistan, and Kyrgyzstan) and encompasses 11 hydrologic basins (Yangtze, Si, Song Hong, Irrawaddy, Hwang Ho, Ganges-Brahmaputra, Indus, Tarim, Ili, Amu Darya, and Syr Darya). HMA, home to over two billion people, experiences warming at a rate that is double the global average (0.32 °C per decade compared with the global average of 0.16 °C per decade) making it one of Earth’s most vulnerable water towers^1^. Warming in HMA has increased precipitation and decreased snowpack and glaciers, which significantly impact water availability downstream^1–3^. In addition to these climatic factors, HMA hosts the largest user of groundwater on Earth, India. As a result, significant decreases in groundwater due to agricultural practices have been documented in the region^4–6^. The unprecedented changing climate along with human footprints have caused the vegetation to rapidly change^7–10^. Indeed, HMA experiences one of the highest greening rates on Earth, which alters the water and energy balances^7,10^. In HMA, the moisture-induced greening triggered by irrigation, decreases in snow, and increases in precipitation and irrigation affect the surface albedo, the ratio of the solar radiation reflected from the Earth’s surface to the solar radiation incident upon it, with consequent impacts on the climate system^11^. Moreover, changes in the precipitation phase (i.e., more precipitation is falling in the form of rain than snow), decreases in the snowpack, and increases in precipitation are shifting the dynamics and the seasonality of the land surface processes with dramatic consequences on water management and hazards^12^.

Understanding the changes in water budgets in HMA and their drivers is essential for water management and climate change mitigation strategies. Nonetheless, because of HMA’s intricate terrain, complex interplay of climate dynamics, and prevalence of human management, the quantification of these changes remains very challenging. First, an accurate representation of the atmospheric dynamics of the region is difficult to undertake because of the harshness of HMA’s environment, which is not easily accessible due to the complex orographic patterns and its high elevation^13,14^. As a result, climate dynamics are poorly constrained, and available meteorological products have large uncertainties. Second, the spatiotemporal variations of HMA’s human footprints are difficult to reproduce because of the lack of reference datasets^15^. For example, accurate estimates of the spatiotemporal variations of irrigation and pumping are not available. Hence, most of the widely used land surface models do not include human management^16,17^. Third, the lack and/or the limited amount of available ground measurements of hydrologic variables make the model evaluation and comparison difficult to perform, making some uncertainties irreducible.

In this study, we rely on multiple remote sensing-based datasets to reduce these uncertainties and better represent the water budget in HMA over the past two decades from 2003 to 2020. We aim to provide estimates of water and energy storage and fluxes that reflect the processes occurring in HMA such as greening and irrigation-driven groundwater depletion as well as their effects on the hydrologic processes. First, we developed ensemble consensus precipitation estimates^14^ using the Integrated Multi-satellitE Retrievals for Global Precipitation Measurement IMERG^18^, the Climate Hazards group Infrared Precipitation with Stations CHIRPS^19^, and the ECMWF Reanalysis ERA5^20^, which were blended by using the probability matched method^21^. We selected these products after comparing the averages and trends of seven widely used gridded precipitation products in the region^14^. The trends and averages of the selected products are consistent with observed hydrological states. Then, we assimilated five different variables into the land surface model Noah-MultiParameterization (Noah-MP^22^) to account for anthropogenic activities and natural changes such as greening and irrigation-driven groundwater depletion in the region and better constrain our model. Because these changes are not driven by atmospheric dynamics, the assimilation of observed variables is required to include them. The 5 assimilated variables are the following:

- (1) Irrigation: which is essential to reproducing groundwater depletion and key land surface changes such as greening. We assimilated spatiotemporal values of irrigation derived from remote sensing datasets^23^.
- (2) Soil moisture (SM): in HMA, changes in soil moisture are triggered by irrigation, increases in precipitation, and decreases in snow cover, therefore, accurate estimates of soil moisture allow for capturing both the impacts of a changing climate and agricultural activities. We assimilated the European Space Agency Climate Change Initiative (ESA CCI) soil moisture^24^.
- (3) Leaf Area Index (LAI): changes in LAI are the primary indicators of greening. Incorporating these changes allows us to not only better reproduce the vegetation dynamics but also capture the hydrodynamics of agricultural lands. We assimilated LAI estimates from the Moderate Resolution Imaging Spectroradiometer (MODIS LAI^25^) instrument aboard the Terra and Aqua satellites.
- (4) Snow Water Equivalent (SWE): cryospheric processes play a significant role in the hydrodynamics of HMA. Nonetheless, the representation of snow dynamics remains challenging, and the land surface models fail to accurately reproduce changes in the snow in the region despite the use of the best available precipitation datasets. Here, we assimilated a remote sensing-based snow water equivalent (SWE) reconstruction which has been tested and evaluated in previous studies to improve the modeled changes in SWE^2^.
- (5) Terrestrial Water Storage (TWS): TWS as provided by the Gravity Recovery And Climate Experiment (GRACE^26^) includes all types of water stored above and below the ground surface, such as snow, ice, groundwater, and surface water storage. GRACE provides a unique opportunity to include measured changes in water and better represent anthropogenic activities such as pumping as well as some processes not included in land surface models, such as changes in glaciers.

The HMA reanalysis based on multivariate data assimilation (MVDA) has allowed a better representation of the land surface processes (i.e., reproduces processes such as greening and irrigation-driven groundwater depletion as well as their effects on the hydrology) in HMA and provides spatially (at 5 km resolution) and temporally (daily) consistent estimates of storages, fluxes, and meteorological conditions including snow (depth, water equivalent), snow extent and albedo, skin/snow/ice temperature, soil moisture, evapotranspiration, groundwater storage, and streamflow that are relevant for a range of model applications including interdisciplinary research and policy development efforts.

## Methods

### Study area: High Mountain Asia (HMA)

**Fig. 1:**
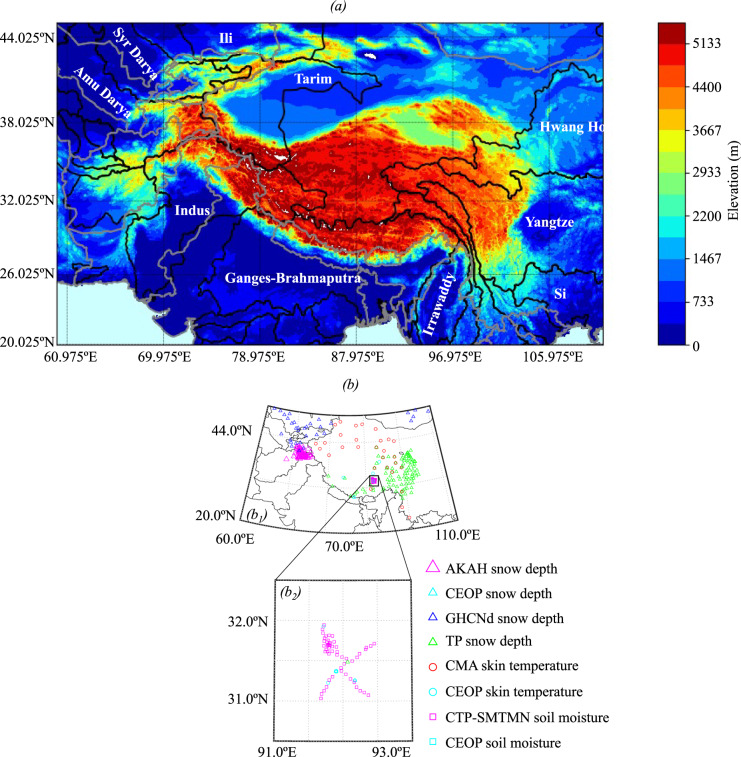
(**a**) Elevation and basins of High Mountain Asia. The gray lines are the country borders, and the black lines are the basin boundaries. (b_1_) Stations used in the evaluations against *in-situ* measurements. (b_2_) A close-up view of stations bounded between 30.5°N and 32.5°N, and 91°E and 93°E.

Our HMA domain extends from approximately 20°N to 46°N, and 60°E to 111°E and hosts the headwaters of Asia’s most prominent hydrologic basins (Fig. 1). These rivers play a crucial role in the downstream hydrology, energy, and economy of countries such as China, India, Pakistan, and Bangladesh. The region is characterized by complex orographic patterns; the elevation varies from sea level to the world’s highest point at Mount Everest (>8000 m), and includes mountain ranges such as the Himalayas, Hindu Kush, Karakoram, Pamir, and Tien Shan and the Tibetan Plateau. The vegetation is very heterogeneous in the region with forests (evergreen and mixed forests) covering the eastern part corresponding to the Yangtze, Irrawaddy, Song Hong, and Si transboundary river basins, crops mostly found over the Ganges-Brahmaputra, Indus, Syr Darya, and Amu Darya basins, grasslands, and shrublands mainly located over the Hwang Ho basin and bare soil which is the dominant land cover of the Tibetan plateau. Because of its complex topography, HMA climate includes both East Asian and South Asian monsoons and the westerlies; as a result, precipitation patterns are highly variable in the region.

### Development of the land reanalysis

The HMA land reanalysis described here leverages an unprecedented set of available remote sensing measurements from several satellites (optical, thermal, passive microwave, laser, and gravity) and has a spatial resolution of 5 km at a daily interval. The land reanalysis was developed from 2003 to 2020, a time frame selected based on the common availability of different remote sensing datasets. In addition to the land reanalysis, an open-loop (OL) simulation (i.e., a simulation without any data assimilation) was also performed to evaluate the advantages of the MVDA.

#### Land surface model set-up

We use the Noah-MultiParameterization (Noah-MP) land surface model version 4.0.1^22^ to develop the land reanalysis. Noah-MP physically simulates key land surface processes and land-atmosphere interactions and has been widely used in the community^22^. The version Noah-MP represents the next generation of the Noah land surface model and incorporates extensive upgrades including dynamic vegetation phenology, a carbon budget and carbon-based photosynthesis, an explicit vegetation canopy layer, and the addition of an unconfined groundwater aquifer. Compared to the Noah land surface model, Noah-MP allows a representation of irrigation processes and groundwater withdrawals and is therefore suitable for land surface modeling over HMA. The model simulates the energy balance at (1) the canopy layer by using a two-stream radiation transfer approach along with shading effects^27,28^ and (2) the ground surface. The vegetation dynamics are represented following Dickinson’s *et al*. approach^29^ and a Ball-Berry photosynthesis-based stomatal resistance^30^. The hydrodynamics are simulated at the soil level characterized by a thickness of 2 m divided into four layers by relying on the Richards equation^31^ and the groundwater by using the TOPMODEL approach. The groundwater is represented by an unconfined aquifer located below the soil column. The temporal variation of the groundwater storage in the unconfined aquifer is equal to the difference between the recharge rate calculated using Darcy’s law and the discharge. Groundwater discharge and surface runoff are simulated using the TOPMODEL approach which consists of expressing these terms as exponential functions of the water table depth^22,32,33^. The water table depth is converted from the aquifer water storage by using the specific yield, which is a constant equal to 0.2. To simulate the spatiotemporal variations of streamflow, we use the hydrological modeling and analysis platform (HyMAP^34^). HyMAP is a global-scale flow routing scheme designed to be coupled with land surface models such as Noah-MP. The model simulates horizontal water fluxes over continental surfaces where the runoff and baseflow generated by Noah-MP are routed through a prescribed river network to oceans or inland seas. HyMAP provides water level, discharge, and storage in rivers and floodplains^34^.

The simulations were performed by using an ensemble precipitation dataset generated by Maina *et al*.^14^ using a localized probability matched method^21^ with three gridded precipitation products (the Integrated Multi-satellitE Retrievals for Global Precipitation Measurement IMERG^18^, the Climate Hazards group Infrared Precipitation with Stations CHIRPS^19^, and the ECMWF Reanalysis ERA5^20^). The ensemble precipitation has been shown to provide consistent and reliable estimates of precipitation and its emerging trends^14,35^. Additionally, surface meteorology fields using downscaled ERA5 temperature, shortwave, and longwave radiation, wind speed, air pressure, and relative humidity^36,37^ are used to force the model runs. We selected ERA5 meteorological forcing because the trends of ERA5 variables such as air temperature are consistent with observed warming in HMA^1^. The model uses high-resolution datasets of elevation, slope, and aspect derived from MERIT-DEM (Multi Error Removed Improved Terrain Digital Elevation Model^38^), the land cover data derived from MODIS^39^ at a resolution of 500 m, soil types derived from the International Soil Reference and Information Centre^40^ at 250 m resolution. The initial conditions for the model simulations are generated by running the model twice from 1990 to 2018 and reinitializing it in 2001 for the MVDA and 1990 for the OL.

#### Multivariate Data Assimilation (MVDA)

We assimilated five variables: (1) remotely sensed applied irrigated water^41^ (2) soil moisture provided by ESA CCI^24^, (3) LAI provided by MODIS^25^, (4) a SWE reconstruction based on the ECMWF Reanalysis version 5 (ERA5) forcing and the MODIS snow cover^42^, and (5) the TWS provided by the GRACE GSFC mascons^26^. As indicated in Table 1, the applied irrigated water was directly added to the model as a source of water (which is extracted from the groundwater), and the assimilation of LAI, SM, and SWE was based on the one-dimensional ensemble Kalman Filter algorithm (EnKF^43^). EnKF is an optimal sequential data assimilation method for nonlinear dynamics that specifically accounts for observation errors and has been widely used to assimilate remotely sensed variables such as LAI, SM, and SWE into the land surface model Noah-MP^44–48^. GRACE TWS was assimilated using the one-dimensional ensemble Kalman smoother (EnKS) because it provides a time-averaged value (over a month). Contrary to EnKF which updates variables whenever an observation is available, EnKS uses information from a series of observations to update model state variables over a window of time and is, therefore, more suitable to datasets such as GRACE.

- **Applied Irrigated Water**
  The Indus and the Ganges-Brahmaputra basins are subject to intense agricultural activities with high rates of irrigation and pumping. We assimilated the spatiotemporal values of applied irrigated water generated by Zhou *et al*.^23^. This dataset is produced by combining a static irrigation dataset the Global Irrigated Area Map (GIAM) and a time-varying irrigation map for India^49^ which is generated by combining yearly MODIS - Normalized Difference Vegetation Index (NDVI) data, Indian Remote Sensing Land Use and Land Cover data, and vegetation condition index data. We directly added this estimated applied irrigated water as a source in the model using the sprinkler irrigation scheme. We assumed that the applied irrigated water originates from groundwater as previous studies have demonstrated that irrigated water in HMA mostly originates from groundwater which explains the high decline of groundwater in India. Following Nie *et al*.^50^ the irrigation scheme implanted in Noah-MP subtracts the groundwater irrigation amount from the model’s groundwater storage term, and the water table depth and groundwater storage are updated accordingly.
- **Leaf Area Index (LAI)**
  Because greening processes are prevalent over HMA, the assimilation of LAI is essential to better incorporating the subsequent changes in land surface processes which have strong implications for hydrology and the energy balance. Moreover, LAI is a key component for calculating evapotranspiration (ET) and gross primary production. Prior studies have shown that the assimilation of LAI leads to improved simulation of the water budget and the representation of hydrodynamics in irrigated lands^51^. We assimilated the LAI values provided by MCD15A2H Version 6 of MODIS^36^ at a spatial resolution of 500 m and a temporal resolution equal to 8 days using the methodology proposed by Kumar *et al*.^51^ based on the EnKF algorithm. In this assimilation framework, the updated LAI from assimilation is used to update the leaf biomass by dividing the LAI value with the specific leaf area, which varies with vegetation type, consistent with the Noah-MP physics formulations^52^. However, the other vegetation mass prognostic variables in Noah-MP related to the stem, wood, and root mass are not updated as part of the assimilation. For the estimation of the uncertainties of MODIS LAI, an additive perturbation with a standard deviation of 0.01 and a temporal correlation of 1 hour was used for both the modeled and the observed LAI (Table 2).

**Table 1:** Assimilated data. EnKF is for Ensemble Kalman Filter and EnKS is for Ensemble Kalman Smoother.

Dataset	Variable	Algorithm
Irrigation	Applied irrigated Water	Direct insertion
ESA CCI	Soil Moisture	EnKF
MODIS	Leaf Area Index	EnKF
SWE	Snow Water Equivalent	EnKF
GRACE	Terrestrial Water Storages	EnKS

**Table 2:** Applied perturbations for the multivariate data assimilation. A is for additive perturbation and M for multiplicative perturbation.

Variables	Type	Standard Deviation	Temporal Correlation	Perturbation cross-correlations
**Forcing**				
Precipitation	M	0.2	24 h	1.0 -0.5 -0.8
Shortwave Radiation	M	30	24 h	-0.5 1.0 0.5
Longwave Radiation	A	0.50	24 h	-0.8 0.5 1.0
**Assimilation of Soil Moisture**				
ESA CCI Soil Moisture	A	0.02	12 h	
Modeled Soil Moisture Layer 1	A	0.1	3 h	1.0 0.6 0.4 0.2
Modeled Soil Moisture Layer 2	A	0.1	3 h	0.6 1.0 0.6 0.4
Modeled Soil Moisture Layer 3	A	0.1	3 h	0.4 0.6 1.0 0.6
Modeled Soil Moisture Layer 4	A	0.1	3 h	0.2 0.4 0.6 1.0
**Assimilation of LAI**				
MODIS LAI	A	0.01	1 h	
Modeled LAI	A	0.01	1 h	
**Assimilation of SWE**				
SWE Reconstruction	M	0.05	3 h	
Modeled Snow Depth	M	0.01	3 h	1.0 0.9
Modeled SWE	M	0.01	3 h	0.9 1.0
**Assimilation of GRACE**				
GRACE TWS	A	5.0	24 h	
Modeled Soil Moisture Layer 1	A	0.005	3 h	1.0 0.6 0.4 0.2 0.0 0.0
Modeled Soil Moisture Layer 2	A	0.005	3 h	0.6 1.0 0.6 0.4 0.0 0.0
Modeled Soil Moisture Layer 3	A	0.005	3 h	0.4 0.6 1.0 0.6. 0.0 0.0
Modeled Soil Moisture Layer 4	A	0.005	3 h	0.2 0.4 0.6 1.0 0.0 0.0
Modeled Groundwater Storage	A	0.1	3 h	0.0 0.0 0.0 0.0 0.0 1.0 0.0
Modeled SWE	M	0.001	30 min	0.0 0.0 0.0 0.0 0.0 0.0 1.0
- **Snow Water Equivalent (SWE)**
  We assimilated the remote-sensing based SWE reconstruction data^2^, which employs a temperature index melt model^53^ along with ERA5 forcing, and MODIS snow cover^42^. We follow the assimilation of snow variables based on the one-dimensional EnKF approach^51^. Similar to the approach in the previous study^51^, the modeled SWE and snow depth were perturbed with a multiplicative noise of 0.01 to represent the uncertainties of the assimilated datasets. A cross-correlation with a coefficient of 0.9 was set for the modeled SWE and snow depth. The observed SWE was perturbed with a multiplicative noise of 0.05.
- **Soil Moisture**
  We used the ESA CCI soil moisture dataset, which provides multi-decadal, global satellite-observed soil moisture estimates starting from 1978 at a temporal resolution of a day and a spatial resolution of 0.25°^24^. The ESA CCI SM v05.2 consists of three surface soil moisture datasets. The product uses single sensors with different instrument characteristics (frequency, spatial resolution, temporal coverage, polarization, etc.) from active and passive microwaves, which have a long legacy for measuring near surface soil moisture, to generate three products: active microwave based only, passive microwave based only, and combined, generated by blending the soil moisture retrievals from active and passive microwave remote sensing instruments. In this study, we assimilated the combined dataset using the one-dimensional EnKF. For the assimilation of soil moisture, the observations are rescaled to the model climatology using the cumulative density function (CDF). The CDFs are derived separately from both the ESA CCI soil moisture retrievals and the soil moisture simulated by Noah-MP at each grid point during the entire simulation period following Kumar *et al*.^44,54^. The uncertainties associated with the soil moisture accounted for in the assimilation procedure are shown in Table 2.
- **Terrestrial Water Storage (TWS)**
  Because of the high decreases in TWS caused by agricultural activities observed in the Ganges-Brahmaputra and the Indus basins, the assimilation of GRACE TWS is important since these processes cannot be represented in the open loop configuration. GRACE provides monthly estimates of the mass variations on the Earth’s surface, which have been used to infer changes in TWS^26,55,56^. In data scarce regions such as HMA, GRACE is often the only available dataset that can be used to better represent water storage. GRACE solutions, however, are coarse in space (on the order of 150,000 km^2^ or larger) and time (monthly). Nevertheless, they can be used to provide an overall constraint on the mass changes, as demonstrated through modeling and data assimilation studies over the world^57–60^. We used the GSFC mascon product, which has a resolution of 0.5°. To assimilate GRACE TWS observations into the land surface model Noah-MP, we used the EnKS as described in Zaitchik *et al*.^57^ and Kumar *et al*.^58^. EnKS is similar to EnKF, where the estimated variables are corrected based on the model and observation uncertainties described by the error covariances. In EnKS, the assimilation increments are computed over certain periods that correspond to the calendar months, and then the smoothing approach temporally disaggregates the increments into the land surface model temporal scale. Therefore, GRACE TWS observations are assimilated into the model at the monthly scale, whenever the observation is available. The assimilation of GRACE TWS was performed in two iterations for each month. The EnKS first generates the model predicted TWS observations by averaging simulated TWS. These predictions are then used to calculate the assimilation increments for the month. Next, the second iteration consists of applying these increments. Irrigation is applied during both the first and second iterations to account for groundwater withdrawal for irrigation in the calculation of TWS.
  The Noah-MP prognostic variables soil moisture, groundwater storage, and SWE were perturbed with additive noise equal to 0.005, 0.1, and 0.001 respectively (Table 2). A correlation length of 3 hours was used for groundwater and soil moisture which is divided into four layers that are cross correlated. The correlation length of SWE is equal to 30 min. An additive perturbation of 5.0 was added to the observed GRACE TWS which has a correlation length of 24 hours to represent the uncertainties of the dataset.
- **Multivariate Data Assimilation (MVDA)**

A model ensemble (of size 20) was created by applying small perturbations to the meteorological forcing inputs and the model prognostic state variables to represent the model uncertainties. Note that unphysical perturbed values were discarded after a quality check. If the perturbations or the analysis updates lead to unphysical values, the corresponding ensemble members are rescaled to valid values using the majority of the valid ensemble members. Similar to the previous studies^51^, we added perturbations to the precipitation, downward shortwave, and longwave radiation at an hourly time step. The perturbations of the forcing include cross-correlations^44^. We used a multiplicative perturbation with a mean of 1 and a standard deviation of 0.5 for the precipitation field. A multiplicative perturbation was also used for the downward shortwave radiation, the mean is equal to 1 and the standard deviation 0.2. Air temperature and downward longwave radiation were perturbed using additive perturbation with a standard deviation equal to 0.5° and 30.0 W/m^2^ respectively. The selected perturbations are shown in Table 2.

## Data Records

The HMA land reanalysis dataset covers the period from 2003 to 2020 and is publicly available on the Natinal Snow and Ice Data Center^61^. The dataset has a spatial resolution of 5 km and a temporal resolution of a day. The reanalysis provides spatially and temporally consistent estimates of storages, fluxes, and meteorological conditions including snow (depth, water equivalent), skin/snow/ice temperature, soil moisture, evapotranspiration, groundwater storage, and streamflow that are relevant for a range of model applications.

## Technical Validation

Despite the importance and vulnerability of mountainous regions, there are few reliable ground measurements because of the complexity, harshness, and diversity of the HMA environment. Monitoring these ground measurements is also expensive and logistically challenging. Hence, the few available *in situ* measurements are sparse in both time and space, which makes model evaluation and validation very challenging. Besides, since most of the available remote sensing observations were already assimilated into the reanalysis, finding independent reference data becomes more difficult. Here we compared the results of our model estimates (ET, snow variables, soil moisture, runoff, streamflow, and groundwater storage) to limited *in situ* measurements in addition to data derived from satellite observations and reanalyses that have not been assimilated (e.g., MODIS skin temperature product).

We evaluated the trends in the simulated variables by comparing them with remotely sensed and measured trends. This reanalysis aims to capture significant changes and dynamics occurring over the HMA such as greening and irrigation-driven groundwater depletion as well as their impacts on the hydrology. To evaluate the ability of MVDA to reproduce these processes, it is important to analyze the trends in the variables of interest. For example, an increasing trend in LAI implies that greening is represented whereas groundwater depletion induced by irrigation will be translated into a decreasing trend in groundwater storage and TWS. All the trends were computed over the reanalysis period from 2003 to 2020 using the Mann-Kendall test which determines whether a time series has a monotonic upward or downward trend^41,62–65^. The Mann-Kendall test uses the following statistics:

1 $$S={\sum }_{i=1}^{n-1}{\sum }_{j=k+1}^{n}\;{sign}({x}_{j}-{x}_{i})$$

where x is the time series variable. The subscripts j and k are the observation time. ${sign}\left({x}_{j}-{x}_{i}\right)$ is equal to +1, 0, or −1, which means increasing, no, and decreasing trends, respectively. Though our study period is not sufficient enough to analyze long-term trends, here we only analyze statistically significant trends, hereafter called “emerging trends”. Besides, previous studies^4,7^ have analyzed greening and irrigation-driven groundwater depletion using remotely sensed datasets which are available to a similar time frame or less. In this study, we assumed that there is no significant trend in the data at a 95% confidence level (or at a significant level of 5%).

For the *in-situ* measurements, similar to prior studies^36,37^, we implemented first-order criteria to optimize the quality of the dataset, e.g., stations with data records less than 365 days are excluded from the evaluation. Further, if the relative elevation difference between the 5-km model grid cell and the collocated station is greater than 50% (with the ground-based station being the baseline), we removed such stations from the evaluation. It is important to note that some ground measurements do not have information about elevation, which makes it more challenging to alleviate stations’ under-representativeness issues via the implemented first-order criteria. For all ground-based evaluations, both OL and MVDA estimates were evaluated at daily time scales via comparisons against *in-situ* measurements taken by the closest collocated ground-based stations. Goodness-of-fit statistics, including bias, root mean squared error (RMSE), unbiased root mean squared error (ubRMSE), and correlation coefficient (R), are computed in all ground-based evaluations following Xue *et al*.^36,37^.

For the remotely sensed data (such as MODIS skin temperature), the evaluation statistics include bias, RMSE, ubRMSE, R, anomaly R, and Kullback-Leibler Divergence score (KLD). The anomaly R, ranging from −1 to +1, is the correlation coefficient between the anomaly time series (i.e., actual values minus climatological means) obtained from model estimates and reference products. The KLD is an information theory-based metric that ranges from 0 to infinity. It measures the difference between an estimated and a reference probability distribution. A KLD equal to 0 indicates that the two compared distributions have identical quantities of information, whereas a larger KLD score indicates a higher degree of difference between the two probability distributions.

### Analysis of the averages and dynamics

#### Annual averages

**Fig. 2:**
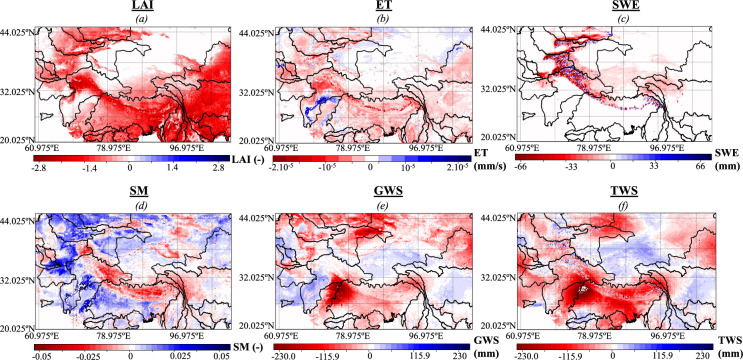
Differences in annual averages of (**a**) Leaf Area Index (LAI), (**b**) evapotranspiration (ET), (**c**) Snow Water Equivalent (SWE), (**d**) Soil Moisture (SM), (**e**) Groundwater Storage (GWS), and (**f**) Terrestrial Water Storage (TWS) between the open loop (OL) and the multivariate assimilation (MVDA). Red indicates areas where OL has higher values than MVDA, whereas areas where OL has lower values than MVDA are colored in blue.

We first compare LAI, ET, SWE, soil moisture, and TWS obtained with MVDA and OL. While OL accounts solely for the hydrological dynamics due to the changes in the climate such as increasing temperature and precipitation, MVDA not only accounts for these effects but also the impacts of human management. Comparing these two simulations will enable the evaluation of the impacts of human activities on hydrology. Figure 2 shows the differences between the annual averages of OL and MVDA. Red indicates areas where OL has higher values than MVDA, whereas areas where OL has lower values than MVDA are colored in blue. Overall, OL has higher values of LAI than MVDA due to the initialization of the model and the high soil moisture of the OL (Fig. 2a). Because of its high values of LAI, OL has higher values of ET than MVDA in general (Fig. 2b). Nonetheless, over the irrigated lands of the Indus basin, ET is higher in MVDA than in OL because of the applied irrigated water. On the contrary to LAI and ET, the differences in SWE between OL and MVDA are bidirectional (Fig. 2c). MVDA increases the value of SWE over high elevation areas because Noah-MP provides low values of SWE in OL. On the other hand, many mid to low elevation areas and some places located in the inner part of the Tibetan plateau have zero values of SWE in MVDA because the latter has a more localized spatial distribution of snow cover, which is derived from MODIS whereas OL tends to spread the extent of the snow cover (Fig. 2c). For soil moisture, the spatial distributions of the difference between OL and MVDA have both positive and negative values (Fig. 2d). Overall, in most areas, MVDA allows for an increase in soil moisture. Over the irrigated lands of the Ganges-Brahmaputra and the Indus basins and other low elevation areas of these basins, the soil moisture increases in MVDA because of the applied irrigated water. Another explanation for the increase in soil moisture in MVDA is its high values of SWE which lead to increased snowmelt. In high elevation areas of the Ganges-Brahmaputra, the Indus, and the Yangtze basins, the soil moisture has lower values in MVDA than in OL. This likely stems from an inaccurate representation of cold season processes and glacier dynamics because these processes mostly control the changes in soil moisture in the region.

As expected, in many regions, MVDA leads to low values of TWS and GWS, notably over the Ganges-Brahmaputra and the Indus basins (Fig. 2e,f). OL does not incorporate the effects of human activities occurring in the region, such as pumping and irrigation. It is well-known that groundwater pumping significantly decreases the TWS and the GWS in this region^4^, as such, the assimilation of GRACE TWS and irrigation allows a better representation of these processes. We note that in some high-elevation areas characterized by seasonal snow dynamics, MVDA leads to higher TWS than OL because it increases the SWE values. MVDA also leads to both increases and decreases in TWS in the northwestern basins. Over snow-covered areas, this is due to the assimilation of SWE as explained above; in other areas, this is likely due to the assimilation of GRACE TWS, which tends to deplete the groundwater because of agricultural activities, and the SWE because of the misrepresentation of the SWE dynamics. In the southeastern edge of our domain (Irrawaddy, Song Hong, Si, and the low elevation of the Yangtze), TWS has higher values in MVDA than in OL. This is likely because the TWS assimilation corrects the biases stemming from the underestimation of the high precipitation from monsoons. In the Hwang Ho basin, MVDA has lower values of TWS than OL, a possible explanation is that glacier melt is not represented in OL and is accounted for in land reanalysis through the assimilation of TWS.

#### Comparisons with remote sensing data and ground measurements

##### Snow detection and false alarm

**Fig. 3:**
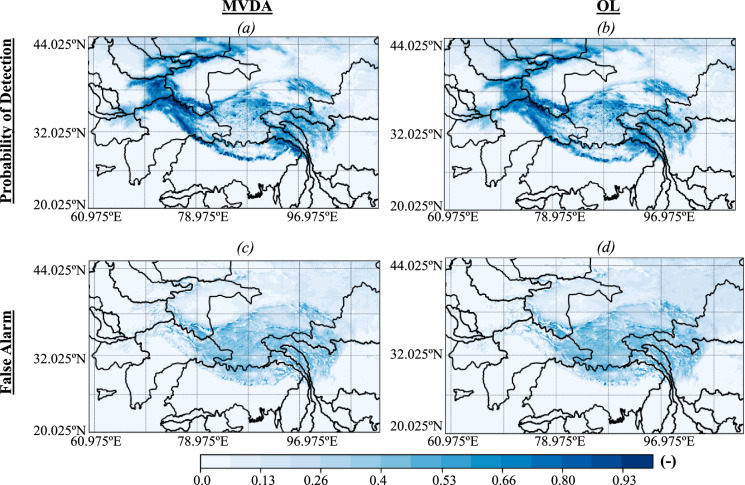
Spatial distributions of the probabilities of detection (annual average) of (**a**) the multivariate assimilation i.e., MVDA and (**b**) the open loop i.e., OL, and false alarm (annual average) of (**c**) MVDA and (**d**) OL compared to the snow cover provided by the Moderate Resolution Imaging Spectroradiometer (MODIS)^49^.

We evaluate the probabilities of detection and false alarm for snow cover of our MVDA and OL products by relying on the snow cover data provided by MODIS^42^. As depicted in Fig. 3, the probability of detection is high (i.e., greater than 75%) over the Himalayas; however, the false alarm ratio reaches 50% in the upper regions of the Himalayas. The probabilities of detection and false alarm of the OL (64% on average for the detection and 35.9% on average for the false alarm) are similar to the ones of the MVDA (67.3% on average for the detection and 32.6% on average for the false alarm), indicating that the MVDA has mostly improved the magnitude of the SWE, and not the spatial extent of the snow cover.

##### Snow depthts

Simulated snow depth were compared against ground-based measurements obtained from 1) the Aga Khan Agency for Habitat (AKAH; https://the.akdn/en/how-we-work/our-agencies/aga-khan-agency-habitat), 2) the Coordinated Enhanced Observing Period (CEOP) Asia Monsoon project (https://www.eol.ucar.edu/projects/ceop/dm/insitu/sites/ceop_ap/), 3) the Global Historical Climatology Network daily database (https://www.ncei.noaa.gov/products/land-based-station/global-historical-climatology-network-daily), and 4) the Observational snow depth dataset of the Tibetan Plateau (TP) from 1990 to 2013 (Version 1.0; https://data.tpdc.ac.cn/en/data/72d6dadf-8e1c-458b-b24e-91539042dfe6/). We used 3 CEOP, 32 GHCNd, 87 AKAH, and 97 TP stations in the snow depth evaluation.

Table 3 summarizes the mean statistics of OL and MVDA estimates against four sets of ground-based snow depth measurements. Overall, the performance of MVDA is superior to OL when compared to GHCNd, AKAH, and TP datasets. However, compared to CEOP snow depth measurements, MVDA yields a degraded performance and OL performs better. Note that this degraded performance is dominated by the evaluation at the CEOP Pyramid station (see Supplementary Figure 1). At the Pyramid site, OL yields a relatively good agreement with CEOP measurements with mean bias = 0.02 m, RMSE = 0.08 m, ubRMSE = 0.07 m, and R = 0.67. However, due to SWE assimilation, a large amount of snowpack is added on top of the OL estimates. As a result, MVDA yields mean bias = 0.70 m, RMSE = 0.74 m, ubRMSE = 0.23 m, and R = 0.29. It is plausible that the assimilated SWE product at 5 km captures the mountain snowpack surrounding the station instead (see Supplementary Figure 1), which forces MVDA related snow estimates to accumulate with a large amount of perennial snowpack. In the evaluation against the 97 TP snow depth measurements, MVDA yields improved skills relative to OL in terms of mean bias (25%), RMSE (15%), and ubRMSE (14%). As TP is featured with an intermittent snowpack (e.g., snow existence with a relatively short duration), redundant OL derived snow can be removed effectively due to SWE assimilation. The most notable difference between MVDA and OL in this set of evaluations can be seen from the interquartile range (IQR), calculated as the difference between the third quartile and the first quartile for each set of goodness-of-fit statistics. The lower the IQR is, the lower the spread is, and the higher the precision is achieved by the corresponding experiment. Relative to OL, the MVDA derived IQR in bias, RMSE, and ubRMSE are improved by 63%, 58%, and 53%, respectively. Further, when comparing model estimates against 87 sets of AKAH snow depth measurements, relative to OL, MVDA improves mean ubRMSE and R by 3% and 13%, respectively. The differences in the performances of MVDA snow depth can be possibly attributed to the 1) scale mismatch between 5 km model grid cell and point-scale (i.e., ~ 10 m by 10 m) measurements, 2) uncertainty in the assimilated SWE product, and 3) *in-situ* snow measurement errors along with missing station descriptions. For example, AKAH does not provide detailed descriptions of the station elevation, which may partly affect the computation of bias and RMSE, and further, yields a less reliable evaluation.

**Table 3:** The computed means of each goodness-of-fit statistics metric, including bias, RMSE, ubRMSE, and R in the evaluation against CEOP, GHCNd, AKAH, and TP snow depth measurements for both open loop (OL) and multivariate data assimilation (MVDA) estimates between 2003 and 2019. n denotes the total number of stations.

Evaluation sources	vs. CEOP snow depth (n = 3)		vs. GHCNd snow depth (n = 32)		vs. AKAH snow depth (n = 87)		vs. TP snow depth (n = 97)	
Model estimates	OL	MVDA	OL	MVDA	OL	MVDA	OL	MVDA
Bias (m)	0.01	0.23	0.06	0.04	0.26	0.32	0.022	0.016
RMSE (m)	0.04	0.26	0.15	0.19	0.40	0.44	0.044	0.037
ubRMSE (m)	0.04	0.09	0.10	0.10	0.30	0.29	0.038	0.033
R (−)	0.61	0.30	0.55	0.55	0.49	0.56	0.23	0.23

##### Soil Moisture

The simulated soil moisture was compared against *in-situ* measurements obtained from 1) CEOP at a 5 cm soil depth, and 2) the Central Tibetan Plateau Soil Moisture and Temperature Monitoring Network (CTP-SMTMN) measuring top-layer soil moisture between 0 cm and 5 cm. There are 4 CEOP stations, and 60 CTP-SMTMN stations after first-order quality controls and screenings.

Table 4 summarizes the mean statistics during the evaluation of OL and MVDA estimates over non-permafrost covered regions against two sources of ground-based soil moisture measurements between 0 and 5 cm. Compared to CTP-SMTMN, MVDA performs better than OL. In the evaluation against the 60 sets of CTP-SMTMN soil moisture measurements, MVDA improves R by ~15% relative to OL and decreases the RMSE and the bias by respectively 0.007 m^3^/m^3^ and 0.008 m^3^/m^3^. Compared to CEOP, MVDA and OL demonstrate the same performance in terms of bias, RMSE, and ubRMSE. Though the evaluation of MVDA snow depth against the CEOP measurements has shown that MVDA performs less than OL, the two simulations have similar performances in terms of soil moisture. Only the R is different between OL and MVDA. These results suggest that the apparent degradation of MVDA when evaluated against CEOP is likely due to the inaccurate location of the station and the mismatch between our model resolution of 5 km and a single point. Indeed, because of the sharp variations of topography in HMA, averaging the snow depth over 5 km can lead to inaccurate estimation of snow depth.

**Table 4:** The computed means of each goodness-of-fit statistics metric, including bias, RMSE, ubRMSE, and R in the evaluation against CEOP 5 cm soil moisture, and CTP-SMTMN top-layer (0–5 cm) soil moisture measurements for both open loop (OL) and multivariate data assimilation (MVDA) estimates between 2003 and 2019. n denotes the total number of stations.

Evaluation sources	vs. CEOP soil moisture (n = 4)		vs. CTP-SMTMN soil moisture (n = 60)	
Model estimates	OL	MVDA	OL	MVDA
Bias (m^3^/m^3^)	0.11	0.11	0.057	0.049
RMSE (m^3^/m^3^)	0.15	0.15	0.118	0.111
ubRMSE (m^3^/m^3^)	0.086	0.086	0.086	0.082
R (−)	0.72	0.63	0.59	0.70

##### Skin temperature

The simulated skin temperature was compared against *in-situ* measurements and satellite-derived products. The *in-situ* skin temperature measurements were obtained from 1) the Chinese Meteorological Administration (CMA), namely the Dataset of Daily Climate Data From Chinese Surface Stations for Global Exchange (V3.0) (http://101.200.76.197/en/?r=data/detail&dataCode=SURF_CLI_CHN_MUL_DAY_CES_V3.0), and 2) CEOP. There are 10 CEOP, and 29 CMA stations used in the skin temperature evaluation. We used the MODIS/Terra Land Surface Temperature Daily L3 Global 1-km Grid (MOD11A1, version 6.1^3^) and the MODIS/Aqua Land Surface Temperature Daily L3 Global 1-km Grid (MYD11A1, version 6.1^3^). The simple arithmetic mean of both nighttime and daytime land surface temperature maps generated by MOD11A1 and MYD11A1 is computed as the reference satellite-based skin temperature measurements following Xue *et al*.^37^.

**Fig. 4:**
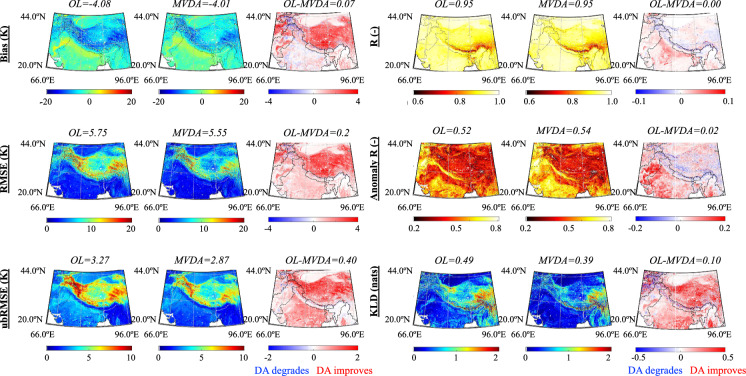
Spatial distribution of bias, RMSE, ubRMSE, R, anomaly R, and Kullback–Leibler divergence score (KLD) computed between daily-averaged, 5-km Open Loop (OL, column 1), multivariate assimilation (MVDA, column 2) surface temperature and MODIS derived surface temperature from 2003 to 2019. Spatial distribution of the change in the absolute value of bias, RMSE, ubRMSE, R, anomaly R, and KLD between OL and MVDA are shown in column 3. The red colors in (**c**), (**f**), (**i**), (**l**), (**o**), and (**r**) indicate MVDA derived estimates agree better with MODIS derived estimates than OL. Conversely, blue colors indicate that OL agrees better with MODIS. The title demonstrates the spatial mean, m, computed for each map.

The skin temperature evaluations against MODIS shown in Fig. 4 demonstrate that, in general, MVDA yields an improved skill relative to OL. For example, the bias, RMSE, ubRMSE, and KLD of MVDA are lower than those of OL by 0.07 K, 0.2 K, 0.4 K, and 0.1 respectively. Likewise compared to CEOP and CMA measured skin temperature (Table 5 and supplementary Figure 2), MVDA decreases the RMSE by 27% for CEOP and 13% for CMA.

**Table 5:** The computed means of each goodness-of-fit statistics metric, including bias, RMSE, ubRMSE, and R in the evaluation against CEOP, and CMA skin temperature measurements for both open loop (OL) and multivariate data assimilation (MVDA) estimates between 2003 and 2019. n denotes the total number of stations.

Evaluation sources	vs. CEOP skin temperature (n = 10)		vs. CMA skin temperature (n = 29)	
Model estimates	OL	MVDA	OL	MVDA
Bias (K)	−2.18	−0.29	0.61	1.00
RMSE (K)	6.03	4.42	5.07	4.39
ubRMSE (K)	4.95	3.65	3.89	3.26

### Analysis of emerging trends

Because of the significant changes in the hydrologic dynamics such as irrigation-driven groundwater depletion and greening and their subsequent impacts on the water and energy cycles, a better representation of the trends in the hydrologic variables is essential. Increasing trends in LAI will indicate that greening has been accounted for whereas decreasing trends in TWS will demonstrate the representation of irrigation-driven groundwater depletion. Because these processes i.e., irrigation-driven groundwater depletion and greening are induced by anthropogenic activities, OL usually fails to capture them. In this study, since we rely on remotely sensed datasets that span the past 20 years to reconstruct the hydrology of the region, the trends analyzed here though statistically significant may not reflect the long-term trends of the region, however, they are sufficient enough to identify greening and irrigation-driven groundwater depletion as shown in previous studies^4,7,11^. Because our goal is to detect the ability of MVDA to capture processes such as greening and groundwater depletion, we only compare the signs of the trends, not their magnitudes. We do so also because of the differences in the definitions of the variables of interest (e.g., ET, runoff, and groundwater storage) between our model and the products we used for model evaluation.

#### Simulated emerging trends

##### Emerging trends in LAI and ET

**Fig. 5:**
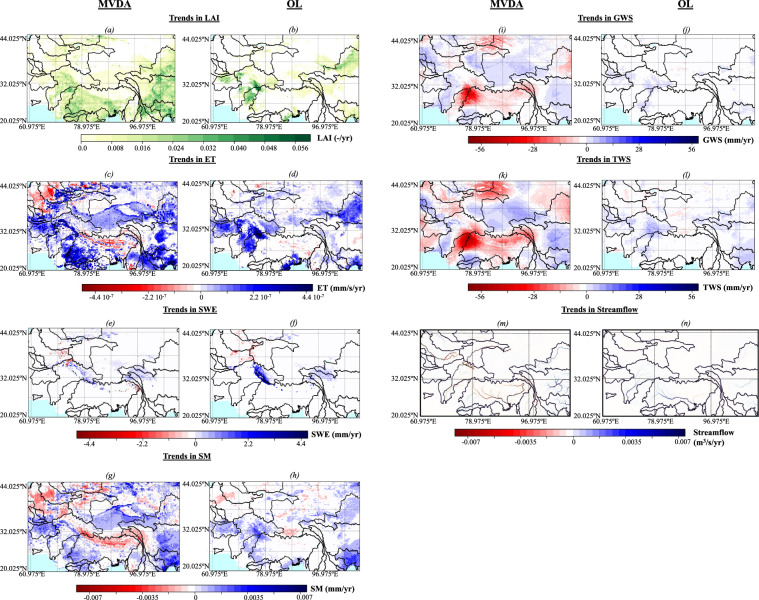
Trends in (**a** for the multivariate assimilation i.e., MVDA and **b** for the open loop i.e., OL) Leaf Area Index (LAI), (**c** for MVDA and **d** for OL) evapotranspiration (ET), (**e** for MVDA and **f** for OL) Snow Water Equivalent (SWE), (**g** for MVDA and **h** for OL) Soil Moisture (SM), (**i** for MVDA and **j** for OL) Groundwater Storage (GWS), (**k** for MVDA and **l** for OL) Terrestrial Water Storage (TWS), and (**m** for MVDA and **n** for OL) streamflow obtained with MVDA (i.e., multivariate assimilation) and OL.

Greening in HMA has been documented in many studies, and recently Maina *et al*.^7^ attributed most of this greening to human activities, notably irrigation, in addition to warming. As a result, the increasing trends in precipitation and temperature incorporated in OL could not solely reproduce HMA’s greening. As illustrated by MODIS LAI observations^25^, greening is occurring almost everywhere in HMA, with the highest increases in LAI over the eastern part of the region corresponding to the Yangtze and the Ganges-Brahmaputra basins (Fig. 5a). These emerging trends in LAI are not captured by OL, where only sparse areas are characterized by an increasing LAI (e.g., the Indus and the Hwang Ho basins) (Fig. 5b). Moreover, these areas do not correspond to the areas with the highest increases in LAI. The latter (e.g., the Ganges-Brahmaputra basin) has unsurprisingly a decreasing trend in LAI. The assimilation of LAI is essential for capturing greening and its impacts on land surface processes such as ET (Fig. 5c,d).

##### Emerging trends in SWE

Both precipitation and temperature are increasing in HMA^12^, however, the increases in precipitation are mainly in the eastern part of the region. In some areas (e.g., the Indus basin), the increase in temperature is not enough to shift the precipitation phase; therefore, the increase in precipitation leads to an increasing SWE despite the warming (Fig. 5e). Decreases in snow are mostly observed in the eastern part of the high elevation areas of the Ganges-Brahmaputra and the western part of the high elevation areas of the Indus corresponding to the western edge of the Karakoram and the Pamir. In the northwestern basins, there is both an increase and a decrease in snow. In general, there is an agreement between the trends in SWE computed with OL and MVDA (Fig. 5e,f). This is because changes in SWE are mostly driven by atmospheric conditions; MVDA only changes the magnitude of the SWE which is low in OL. In general, OL simulation has a more pronounced trend than MVDA.

##### Emerging trends in soil moisture

Both anthropogenic (e.g., irrigation) and climatic (changes in precipitation and temperature) factors affect soil moisture. As detected by the ESA CCI soil moisture, the soil moisture derived from MVDA is generally decreasing in many areas of the region; only the eastern part, dispersed areas of the northwestern basins, and the Ganges-Brahmaputra show an increasing trend (Fig. 5g). Overall, the emerging trends in soil moisture obtained with MVDA are consistent with OL (Fig. 5h). However, the emerging trends in soil moisture in MVDA are more pronounced. In particular, the decrease of soil moisture in the Himalayas is more noteworthy in MVDA than in OL.

##### Emerging trends in TWS

Similar to the patterns in soil moisture, the emerging trends in TWS are influenced by both anthropogenic and climatic factors. TWS resulting from MVDA has a bidirectional trend, with an increasing trend in the eastern edge of HMA subject to the monsoon and the inner portion of the Tibetan Plateau and a decreasing trend in the southern parts of the Indus and the Ganges-Brahmaputra basins because of the overuse of groundwater, the Hwang Ho, and the northwestern basins (Fig. 5k). In the Indus and the Ganges-Brahmaputra basins, OL captures increasing and statistically insignificant trends, respectively, whereas MVDA (like GRACE) depicts a decreasing TWS (Fig. 5k,l). The overuse of groundwater due to agricultural activities occurring in these areas cannot be reproduced by OL. Only the increasing trends in TWS due to an increase in precipitation at the eastern edge are reproduced by OL. Moreover, there are noteworthy decreases in TWS over the Himalayas depicted in MVDA and not in OL; those high changes in TWS are likely due to glacier melt, which is not physically represented in Noah-MP. The Yangtze basin has a bidirectional trend in TWS in MVDA; the high elevation zone is characterized by a decrease, whereas the low elevation zone has an increase in TWS. These spatial patterns of trends are not seen in the OL results. There is likely a source of recharge (e.g., glacier melt depleting the TWS in high elevation areas and increasing it in low elevation) that is not accurately reproduced by OL but depicted in MVDA. A similar behavior is also likely at the origin of the differences in trends between OL (which shows low increases and decreases in TWS) and MVDA (characterized by high decreases in TWS) over the Hwang Ho and the northwestern basins. Another explanation for the decrease in TWS in the Hwang Ho basin is the impact of anthropogenic activities and/or land degradation.

##### Emerging trends in streamflow

Because of the lack of data, we did not assimilate streamflow; however, we expect the different assimilations to affect the streamflow. Figure 5m,n show the emerging trends in streamflow obtained with MVDA and OL. In OL, streamflow is increasing everywhere in the domain due to the increase in precipitation and the decreases in snow that lead to increasing snowmelt (Fig. 5n). However, in MVDA, streamflow decreases in the Ganges-Brahmaputra, the Indus, and the northwestern basins while it increases in the Yangtze and the Hwang Ho (Fig. 5m). Over the Ganges-Brahmaputra basin, pumping decreases the water table depth. As a result, surface water recharges the groundwater, and more precipitation is used to compensate for the decreasing groundwater rather than contributing to the runoff. Because OL depicts an increase in streamflow over the Ganges-Brahmaputra basin, the decrease observed in MVDA is likely not stemming from the changes in precipitation and SWE as these processes are included in OL. Therefore, groundwater pumping is likely the driver of these decreases in streamflow. Likewise, in the Indus basin, streamflow decreases despite increasing precipitation and SWE likely because of groundwater pumping. In OL, the northwestern basins are characterized by low to no-trends in streamflow. In MVDA, however, the streamflow has a pronounced bidirectional trend, though it is decreasing mostly everywhere, likely because of the assimilated decreasing SWE and GRACE TWS. Similarly, in OL very little change in SWE is depicted in the Yangtze and Hwang Ho basins despite the increasing precipitation, contrary to MVDA. As explained above, there is likely glacier melt in the high-elevation zones that has not been considered in the model, recharging the groundwater downstream and impacting the streamflow.

#### Comparisons to trends derived from remotely sensed datasets

**Fig. 6:**
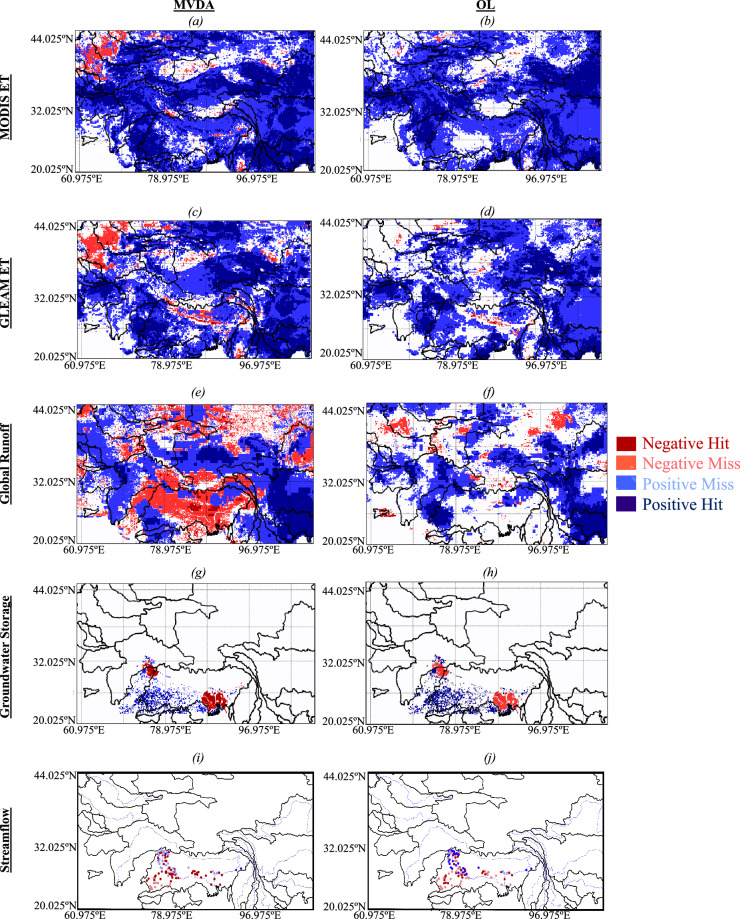
Trends detection for evapotranspiration (ET) compared to (**a** for the multivariate assimilation i.e., MVDA and **b** for the open loop i.e., OL) the Moderate Resolution Imaging Spectroradiometer (MODIS, MOD16^63^) and (**c** for MVDA and **d** for OL) the Global Land Evaporation Amsterdam Model (GLEAM^64^), runoff compared to the global runoff data provided by (Ghiggi *et al*.^65^) (**e** for MVDA and **f** for OL), groundwater storage compared to measurements (**g** for MVDA and **h** for OL), and streamflow compared to measurements (**i** for MVDA and **j** for OL). “Negative Hit” are locations where both remotely sensed and simulated values have negative trends, in “Negative Miss” observations have negative trends and simulations positive, in “Positive Hit” both remotely sensed and simulated values have negative trends, and in “Positive Miss” observations have positive trends and simulations negative.

We compared the trends in the simulated (for both MVDA and OL) ET, runoff, and groundwater to the trends provided by remotely sensed and ground measurements. Figure 6 illustrates the comparisons of the trends in ET obtained with OL and MVDA and remotely sensed ET from MOD16^66^ (Fig. 6a,b) and the Global Land Evaporation Amsterdam Model (GLEAM^67^, Fig. 6c,d). Overall, the trends in simulated ET (obtained with both OL and MVDA) agree with the trends in ET derived from MOD16 and GLEAM. However, the trends obtained with MVDA are closer to the remotely sensed trends (40% on average) than those of the OL (25% on average). For example, in the Yangtze, the increases in ET in MVDA triggered by greening agree with the trends in the remotely sensed products, whereas OL indicates a decrease in ET because it does not account for greening. Likewise, in the Ganges-Brahmaputra basin, the zero and positive trends in ET over the irrigated croplands in MVDA agree with those of MOD16 and GLEAM, while OL, which does not incorporate irrigation practices, indicates a decrease. Nevertheless, both OL and MVDA indicate a decreasing trend in ET in the Himalayas, while MOD16 and GLEAM indicate a positive trend in ET. Such differences between simulated and remotely sensed ET are likely arising from our precipitation forcing dataset, which has a decreasing trend in this region. Another explanation for the decreasing trend in ET is the assimilation of GRACE TWS, which leads to reduced subsurface moisture storage, and, therefore, a reduction in ET^60^.

Overall, 42% of the trends in runoff obtained with MVDA are in agreement with the trends in the global runoff provided by (Ghiggi *et al*.^68^) whereas this percent is only 29% for OL (Fig. 6e,f). Over some low elevation areas of the Ganges-Brahmaputra basin, the decreasing trends in runoff obtained with MVDA are consistent with the global runoff data whereas OL indicates statistically insignificant trends. Over the Himalayas, our MVDA indicates decreasing trends in runoff whereas the global runoff data indicates increasing trends. Such inconsistencies may arise from the differences in meteorological forcing. Simulated runoff obtained with OL and MVDA over the Yangtze and the Irrawaddy basins is consistent with the global runoff data.

Groundwater measurements were mostly available over the Indus and the Ganges-Brahmaputra basins. Overall, these measurements indicate a decreasing trend, this is well captured by MVDA where 80% of the trends agree with the measurements (Fig. 6g,h). On the contrary, OL only captures 26% of the measured trends, these are mostly the increasing trends of groundwater storage located in the southwestern edge of the Ganges-Brahmaputra basin.

Like the groundwater measurements, most of the streamflow data we obtained are over the Ganges-Brahmaputra basin. Streamflow measurements are characterized by decreasing trends, only the stations located in the headwater have positive trends though very small. MVDA allows capturing these decreasing trends notably at the stations located downstream where the negative trends in streamflow are noteworthy. 76% of the negative trends of the measured streamflow are captured by MVDA whereas OL only captures 38% (Fig. 6I,j). In addition to missing the overall decreasing trend in streamflow, OL overestimates peak flow and the streamflow in general compared to MVDA, whose temporal variations of streamflow are closer to the observations (Supplementary Figure 3).

## Usage Notes

The HMA reanalysis contains gridded files with land surface (soil moisture, evapotranspiration and its components, surface albedo, gross primary production, snow depth, snow water equivalent, snow cover, leaf area index, sensible and latent heat flux), surface (runoff and streamflow), and subsurface (groundwater storage, terrestrial water storage) variables written as daily outputs with a resolution of 5-km in netCDF-4 format. The files are publicly available and archived at the National Snow and Ice Data center (NSIDC, https://nsidc.org/data/hma2_nlsmr/versions/1)^61^ Distributed Active Archive Center (DAAC) in compliance with NASA data standards (https://earthdata.nasa.gov).

### Supplementary information

Supplementary Information

## Data Availability

The data were generated using the NASA Land Information System (LIS, http://lis.gsfc.nasa.gov), which is a comprehensive land surface modeling and data assimilation framework that supports modeling over user-specified regional or global domains using an ensemble of land surface models. The code is publicly available on GitHub: https://github.com/NASA-LIS. The datasets used in this study can be found on the following websites: •  ERA5 forcing can be found in https://www.ecmwf.int/en/forecasts/dataset/ecmwf-reanalysis-v5 •  IMERG Precipitation: https://gpm.nasa.gov/taxonomy/term/1372 •  CHIRPS Precipitation: https://www.chc.ucsb.edu/data •  SWE reconstruction by Kraaijenbrink *et al*.^2^: https://zenodo.org/record/4715786#.YqDY0S-B1pI •  MODIS LAI: https://lpdaac.usgs.gov/products/mcd15a2hv006/ •  ESA CCI soil moisture: https://www.esa-soilmoisture-cci.org/data •  GRACE data: https://earth.gsfc.nasa.gov/geo/data/grace-mascons
