# Supplementary material for: Development of a multidecadal land reanalysis over High Mountain Asia
Source: Sci Data. 2024 Jul 27;11:827. doi: 10.1038/s41597-024-03643-z (PMC11283528; doi:10.1038/s41597-024-03643-z)
Supplement: Supplementary file 1 — Supplementary Information [file 41597_2024_3643_MOESM1_ESM.docx]

**Supplementary Information**

Supplementary Figure 1: (a) Time series of open loop (OL) and multivariate data assimilation (MVDA) derived snow depth estimates in the comparisons against the CEOP Pyramid station measured snow depth at (27.959ºN, 86.8132ºE). (b) The CEOP Pyramid station image. (c) The snow depth sensor installed at the CEOP Pyramid station. Figures (b) and (c) are downloaded from <https://archive.eol.ucar.edu/projects/ceop/dm/insitu/sites/ceop_ap/Himalayas/Pyramid/>

Supplementary Figure 2: Scatter density plots of skin temperature measurements at stations CMA and CEOP in comparisons to open loop (OL), multivariate assimilation (MVDA), and MODIS skin temperature estimates between 2003 and 2019.

Supplementary Figure 3: Monthly streamflow time series over the 2000-2020 period at gauges located in the (a) Ganges (at Harding Bridge station) and (b) Brahmaputra (at Bahadurabad River station) Rivers, near the outlet (see their locations in c). The blue line indicates the observations and the red line the results of the multivariate assimilation (MVDA).
